# Baculovirus helper functions during recombinant adeno-associated virus production in insect cells for gene therapy applications

**DOI:** 10.1016/j.omta.2026.201876

**Published:** 2026-10-26

**Authors:** Linda A. de Jong, Monique M. van Oers, Gorben P. Pijlman

**Affiliations:** 1 Laboratory of Virology, Wageningen University & Research, Droevendaalsesteeg 1, 6708 PB Wageningen, the Netherlands

**Keywords:** adeno-associated virus, gene therapy, baculovirus, baculovirus expression vector system, helper virus, viral vectors

## Abstract

Adeno-associated virus (AAV) can only replicate when additional functions are supplied via a co-infection with a helper virus (typically an adenovirus or herpesvirus) or co-transfection with helper virus genes. Use of the baculovirus expression system for recombinant (r)AAV gene therapy vector production in insect cells is becoming more common, although the helper functions provided by the baculovirus have not been identified. We first review the basics of AAV biology and the production of rAAVs in insect cells. We describe adenovirus and herpes simplex 1 virus helper genes to identify functional homologs in the baculovirus genome. Next, we provide a shortlist of candidate baculovirus genes that are likely involved in cell cycle coordination, DNA synthesis, promoter transactivation, and stress response modulation to support AAV replication. We provide experimental approaches to identify direct and/or indirect interactions between baculovirus gene products and AAV during replication, capsid synthesis and genome packaging. This work contributes to our fundamental understanding of baculovirus-AAV interactions, supporting the rational engineering of improved rAAV production in insect cells.

## Graphical abstract

**Figure:**
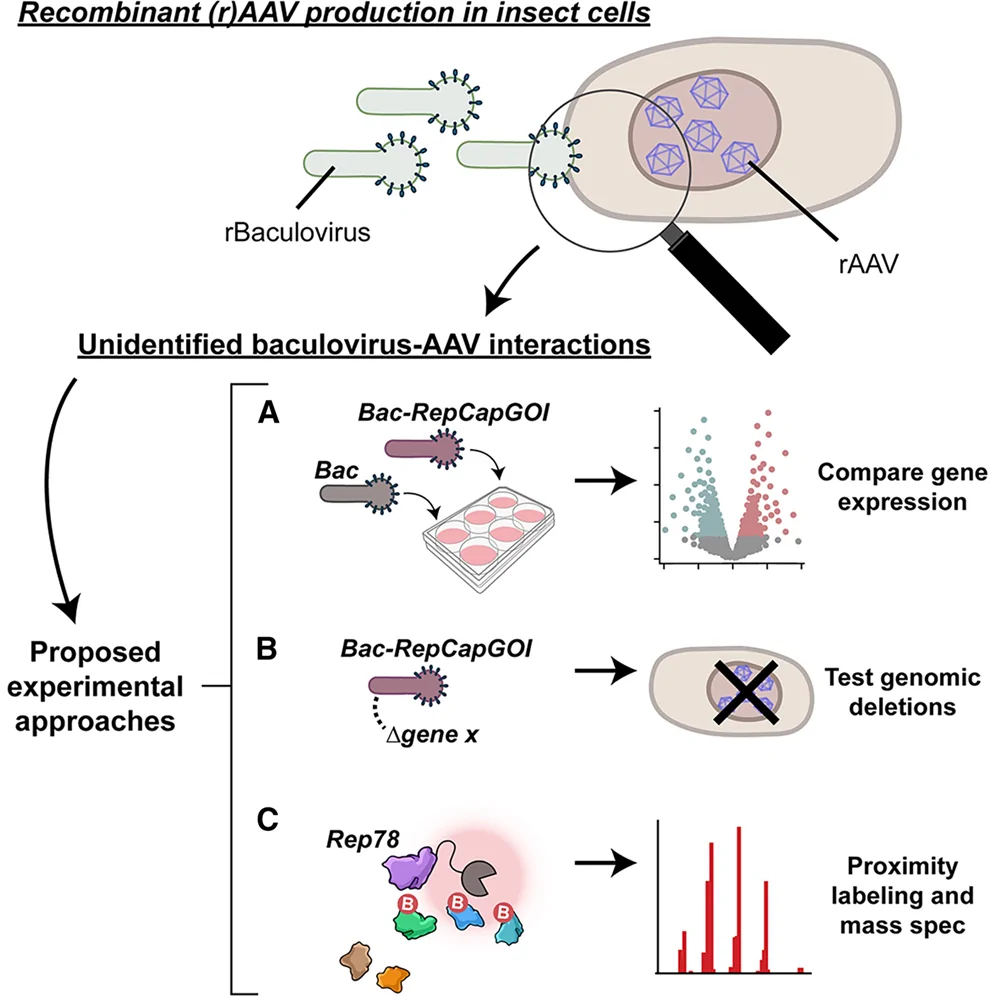

## Teaser

De Jong and colleagues review how baculovirus enables adeno-associated virus replication in insect-cell systems. By identifying candidate helper genes and outlining strategies to dissect their roles, the work advances mechanistic understanding and supports the development of more efficient, scalable gene therapy vector production platforms.

## Introduction

Since adeno-associated virus (AAV) derived vectors have proven to be highly suitable for applications in gene therapy, their efficient production at a large scale has become important. What specifically makes the production of recombinant (r)AAVs challenging is that AAV (family *Parvoviridae*; subfamily *Parvovirinae*, genus *Dependoparvovirus*) cannot complete its replication cycle without specific helper functions being supplied.^1^^,^^2^ Initially, these helper functions were delivered to mammalian cells via co-infection with a known helper virus, most commonly an adenovirus (AdV) or herpes simplex virus (HSV).^1^^,^^3^^,^^4^ To ensure safety of the therapeutic end product, the separation of the co-infecting pathogenic AdV or HSV from the rAAV vectors is necessary, which makes for a complex and expensive downstream processing process. Since then, the rAAV production process has undergone substantial development and the production of rAAV in mammalian cells is mediated by co-transfection with plasmids providing the viral helper elements (AdV5 *VA RNA*, *E2a*, and *E4orf6*) together with the components to assemble the rAAV vector (*Rep*, *Cap*, and a gene of interest [GOI]).^5^^,^^6^^,^^7^ More recently, mammalian producer cell lines have been developed, that stably or inducibly express required AAV genes, thereby simplifying and optimizing the system considerably.^8^^,^^9^ Although the omission of infectious helper viruses from the production process increases the safety, the scalability remains challenging due to the limitations of plasmid DNA transfections compared to helper virus infections.

In the early 2000s, another method for the generation of rAAVs was described based on the baculovirus expression vector system (BEVS) in insect cells.^10^ Enabled by the strong promoters offered by the BEVS, rAAV was produced in *Spodoptera frugiperda 9* (Sf9) cells grown in suspension that were infected with three recombinant baculoviruses, each harboring one of the rAAV components (AAV *Rep*, *Cap* or GOI). In the years that followed, improvements to the BEVS-rAAV system were reported, mainly through increasing the stability of the transgene cassettes and by reducing the number of baculoviruses required for production.^11^^,^^12^ These studies demonstrated that high yield rAAV production with the BEVS in insect cells is possible without AdV or HSV helper functions, and led to the first rAAV product ever that received market authorization by the European Medicines Agency (EMA); Alipogene tiparvovec (Glybera, Uniqure), a gene therapy treatment designed to overcome lipoprotein lipase deficiency.^13^ Since then, the EMA and US Food and Drug Administration (FDA) have approved more rAAV therapeutics that were produced with the BEVS; Valoctocogene roxaparvovec (Roctavian; Biomarin) and Etranacogene dezaparvovec (Hemgenix, UniQure & CSL Behring LLC) for the treatment of hemophilia A and B, respectively.^14^^,^^15^

Little is known about which baculovirus genes, proteins and/or processes play a role in directly supporting AAV replication in insect cells, and/or indirectly providing support by creating a permissive intracellular environment, despite the fact that helper functions have been extensively studied for mammalian helper viruses. Since its first description for rAAV in 2002, multiple experimental and review papers have covered the BEVS as a production platform for rAAVs. Subsequently, this has contributed to progress in process optimization, a better understanding of baculovirus co-infection kinetics, optimization of the *Cap* sequence to achieve the desired capsid protein stoichiometry, as well as the generation of rAAV packaging insect cell lines.^16^^,^^17^^,^^18^ However, despite all these developments important fundamental questions are left unanswered regarding the molecular interactions between baculovirus and AAV replication. There are plausible indirect mechanisms by which baculovirus proteins and the baculovirus-infected cell state provide the helper environment for AAV replication, but the protein interactions have not been well characterized in the literature. A deeper understanding of these interactions might lead to additional process improvements, which in turn might lead to reduction in process costs, all aiding toward making rAAV gene therapies more affordable. In this review, we discuss AAV biology and BEVS-mediated rAAV production, highlight the importance of host cell environment, and nominate baculoviral gene candidates for further study. Lastly, we describe a future experimental workflow to better define these interactions.

## Adeno-associated virus

### Taxonomy

Members of the family *Parvoviridae* are characterized by a small, non-enveloped capsid containing a linear, single-stranded DNA (ssDNA) genome of approximately 4–6 kb.^19^ A defining hallmark of this family is their conserved mechanism of genome replication, known as rolling hairpin replication (RHR).^20^ This replication strategy relies on terminal hairpin structures flanking the coding part of the genome, which serve as self-priming origins of DNA synthesis. While mechanistically related to rolling-circle replication (RCR), used by, for instance, bacteriophages^21^ and geminiviruses,^22^ RHR is distinct in its reliance on linear DNA templates flanked by hairpin termini.^23^ All members of the *Parvoviridae* encode replication initiator proteins that are functionally conserved and responsible for origin recognition, site-specific nicking, and helicase activity.^19^ This conservation spans vertebrate-infecting viruses, including AAV, as well as viruses that infect invertebrate hosts, such as those in the *Densovirinae* sub-family. The members of the *Densovirinae* are able to autonomously replicate in invertebrates, without the need for helper virus functions.^24^ The shared replication logistics across parvoviruses implies that AAV replication might be supported in insect cells, which was eventually validated experimentally.^10^

Taxonomically, AAV belongs to the genus *Dependoparvovirus* in the *Parvovirinae* sub-family, within the *Parvoviridae* family (Figure 1A).^19^ Despite the genus name implying obligate dependence on helper viruses, this requirement is not universal. Autonomously replicating viruses such as goose parvovirus (GPV) and Muscovy duck parvovirus (MDPV) are classified within the same genus yet replicate independently of helper viruses (Figure 1B).^30^^,^^31^^,^^32^ Comparative sequence analyses reveal a substantial similarity of approximately 61% between AAV and GPV genomes, and evolutionary studies suggest that these autonomous and non-autonomous dependoparvoviruses share a common ancestor.^31^^,^^33^ Studies of the AAV life cycle indicate that the block to autonomous replication occurs at multiple discrete stages. In the absence of helper virus functions, AAV is impaired in efficient second-strand DNA synthesis. This also affects the transcription of the viral genome by host RNA polymerase II, preventing expression of genes for replication and subsequent genome replication.^30^^,^^31^^,^^34^ These deficiencies contrast with the GPV and MDPV dependoparvoviruses, which replicate autonomously without this dependance on a helper virus co-infection to replicate.^31^

**Figure 1:**
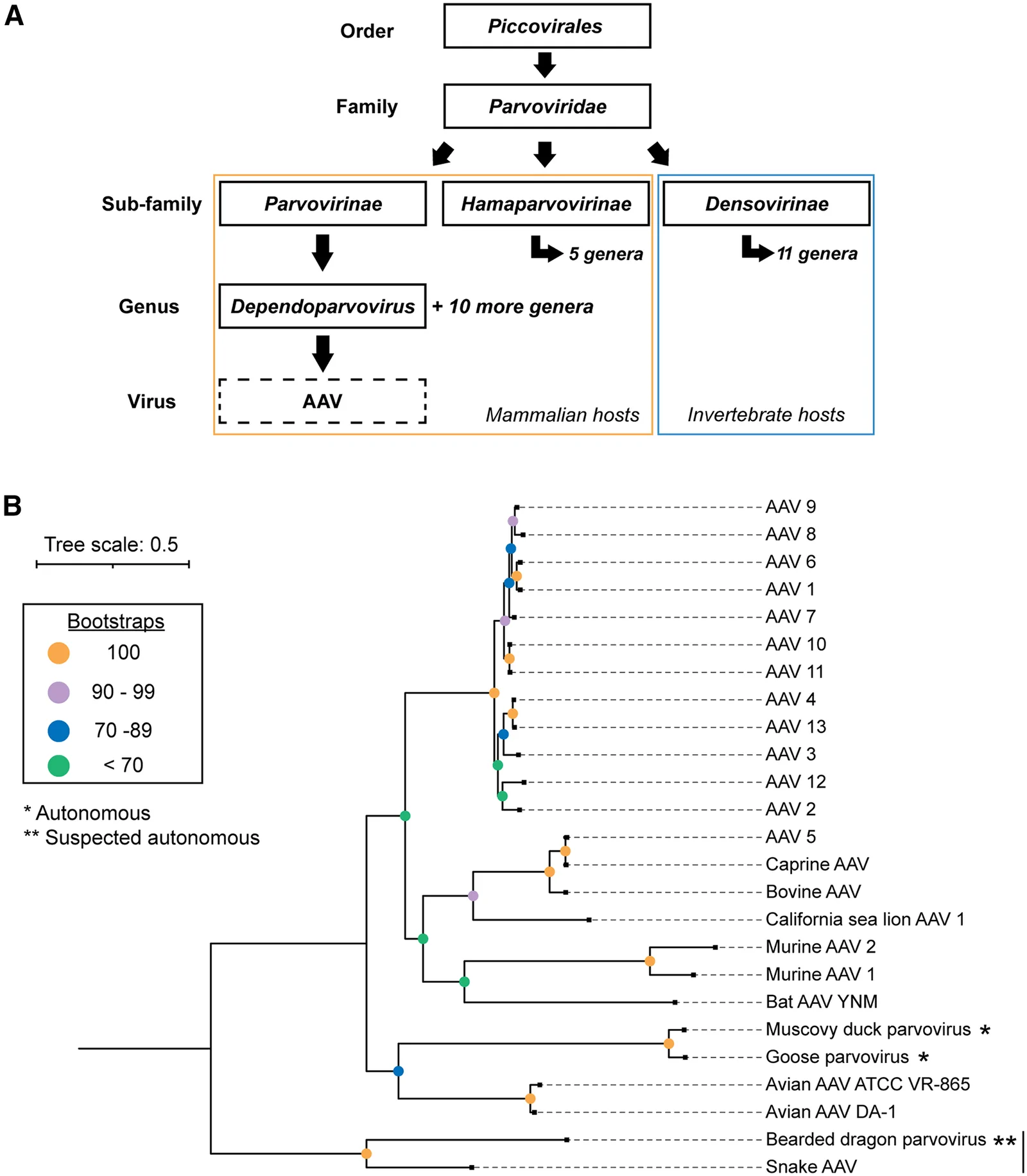
Taxonomy of AAV and a phylogenetic tree of the viruses belonging to the *Dependoparvovirus* genus (A) Taxonomic position of AAV. (B) Phylogenetic tree of circulating dependoparvoviruses based on the alignment of NS1 protein sequences (retrieved from https://www.ncbi.nlm.nih.gov/), and adapted from Hildebrandt et al.^25^ The tree was rooted using an outgroup with two viruses, Bearded dragon parvovirus (genus: *Dependoparvovirus*; species: *Dependoparvovirus* squamate2) and snake parvovirus (genus: *Dependoparvovirus*; species: *Dependoparvovirus* squamate1). The colored dots at the branch nodes represent the bootstrap support values (see legend). Indicated with ⁎ or ⁎⁎ are viruses that are autonomous or suspected to be autonomous, respectively. Methodology: the NS1 protein sequences were retrieved from GenBank (accession numbers provided in Table S1) and analyzed in a Linux-based environment. MAFFT v7.520 was used to perform a multiple sequence alignment (MSA) (-maxiterate 1,000; -localpair).^26^ The alignment was trimmed with trimAI v1.5.0 (-gt 0.6 -w 3),^27^ and the tree was generated with ModelFinder in IQ-TREE v2.3.6 (-B 1,000).^28^ iTOL was used for visualization of the tree.^29^

At the functional level, AAV serotypes exhibit a high degree of modularity. The hairpin-shaped, inverted terminal repeats (ITRs) flanking the rest of the genome, have been shown to be interchangeable between different AAV serotypes, as have genes for the replication proteins, enabling cross-serotype replication and packaging in recombinant systems.^35^^,^^36^ Similarly, the assembly-activating protein (AAP) from AAV2 can support nucleolar targeting and capsid assembly of multiple other serotypes, underscoring strong functional conservation within the genus.^37^ This interchangeability has been exploited in recombinant AAV production platforms, in which *Rep* from one serotype can often be combined with ITRs and capsids from another serotype without loss of functionality.^38^^,^^39^ Notably, AAV serotype 5 (AAV5) forms an exception to this gene function exchange ability, probably because it represents the most divergent serotype within the AAV clade, both genetically and antigenically (Figure 1B).^25^^,^^38^

The evolutionary conserved features such as the modular genome organization hint toward a conserved replication strategy within the *Dependovirus* genus, despite the unique dependence of AAVs on helper virus functions. Understanding how this dependence emerged evolutionarily and how the helper functions can be substituted by diverse heterologous systems such as AdV, HSV, or baculovirus infection remains important for both understanding AAV biology as well as optimization of rAAV vector production.^40^

### Virion structure and genome organization

Most of the reported AAV characterization is based on serotype AAV2, though there are at least thirteen identified AAV serotypes that naturally occur.^41^ The virions are nonenveloped, have an icosahedral shape, and a diameter around 25 nm (Figure 2A). The capsid has a T=1 symmetry and is composed of three viral proteins, VP1, VP2, and VP3, in a stoichiometric ratio of approximately 1:1:10, respectively.^42^ Without a helper virus, AAV can enter the cell, move through the cytoplasm, enter the nucleus and uncoat its genome.^43^ Here the genome persists predominantly in an episomal form as monomeric or concatenated double stranded DNA, or alternatively integrates at low frequency into the host genome.^44^ For wildtype (wt)AAV2 the integration frequency is approximately 1 in 1,000 infectious particles.^45^ Integration mainly occurs in the specific AAVS1 site in the human genome on chromosome nineteen (19q13.3), although it can also occur elsewhere.^46^^,^^47^^,^^48^^,^^49^ Only when essential helper virus genes are present, AAV replication is initiated.

**Figure 2:**
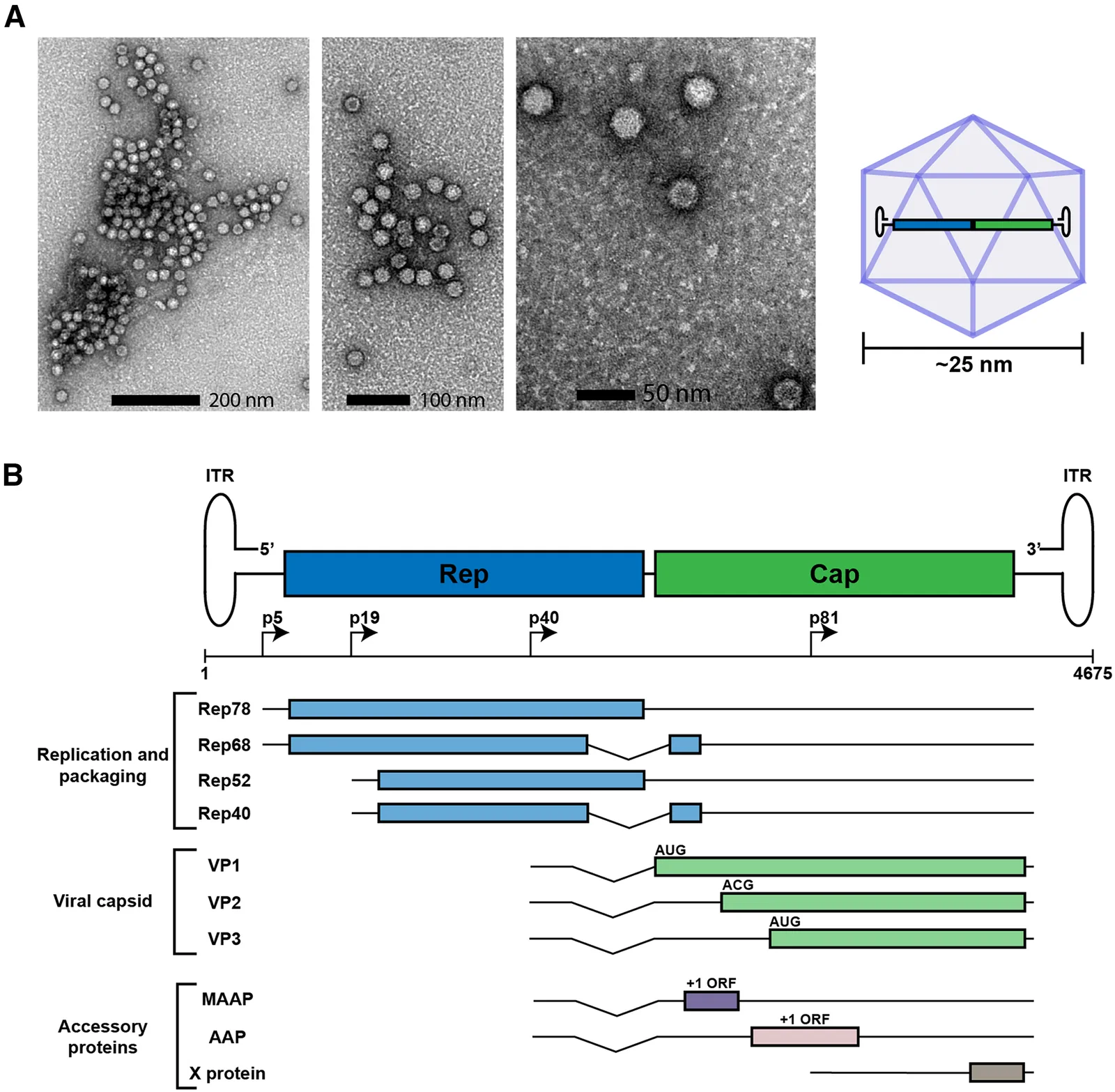
AAV virion structure and genome organization (A) Purified rAAV preparations were stained with 2% uranyl acetate and imaged with transmission electron microscopy (TEM). A schematic view of the virion structure is included on the right. (B) Genome organization of the wt AAV2 genome. The virus has a linear single-stranded (ss) DNA genome with two major orfs, Rep and Cap, flanked by ITRs. Gene expression is mediated from four promoters (p5,p19, p40, and p81) from which mature transcripts originate via alternative splicing. Further protein diversity originates from alternative translation start codons.

The wtAAV2 genome consists of linear, ssDNA with a size of ∼4.7 kb (Figure 2B).^50^ Positive or negative genome strands are packaged with equal efficiency.^2^^,^^51^ The ends of the genome are 145 nt long ITR sequences that partially self-fold into hairpin-like structures.^50^ The ITRs are the only parts of the wt AAV genome that are retained when producing recombinant vectors. The ITRs are important for the initiation of DNA replication, as well as for providing packaging signals to facilitate genome encapsidation, and for mediating DNA integration into the host genome.^52^^,^^53^^,^^54^ The AAV genome contains two major coding regions: *Rep* and *Cap*. The p5 promoter generates an mRNA transcript that is translated into Rep78, and a shorter, spliced variant translating into Rep68. A non-spliced mRNA and a spliced variant produced from the p19 promoter translate into Rep52 and Rep40, respectively. The “major” Rep proteins (Rep78 and Rep68) are mostly involved in genome replication, while the “minor” proteins (Rep52 and Rep40) play a role in genome encapsidation.^55^ When producing rAAVs, supplying only one major and one minor Rep protein, typically Rep78 and Rep52, is sufficient for replication and encapsidation of genomes.^56^ The cap mRNA is transcribed from the p40 promoter, and gives rise to the three capsid proteins (VP1, VP2, and VP3), membrane-associated accessory protein (MAAP) and AAP. The individual capsid proteins VP1, VP2, and VP3 are translated from mRNAs produced via alternative RNA splicing and together assemble into the viral capsid. Translation of overlapping open reading frames (orfs) in the VP1 gene results in the expression of MAAP and AAP.^57^^,^^58^^,^^59^ The accessory protein MAAP was discovered most recently and serves as a viral egress factor aiding in release of AAV particles from the host cell.^60^^,^^61^ AAP stimulates capsid protein transport to the nucleolus, and is involved in capsid assembly.^58^ In addition, a minor coding region near the 3′ end of the cap orf encodes the X protein.^50^ Expression of the *X* gene is regulated by the p81 promoter, and this protein functions as an enhancer for DNA replication and virion production.^62^^,^^63^

## Baculovirus-mediated rAAV production

### Baculovirus biology and expression vector system

Baculoviruses are insect-infecting viruses that are commonly used for pest control purposes,^64^^,^^65^^,^^66^ and are applied in biotechnology for the expression of recombinant proteins, virus-like particle generation and production of rAAVs.^10^^,^^67^^,^^68^^,^^69^ Autographa californica multiple nucleopolyhedrovirus (AcMNPV) has been used as the basis for the development of the BEVS.^70^^,^^71^

AcMNPV, like other viruses in the *Alphabaculovirus* genus, produces two types of progeny virus: budded virus (BV) and occlusion-derived virus (ODV). BVs are generated during the “late phase” of infection by budding of nucleocapsids from the surface of infected cells, after which they facilitate the viral spread throughout an infected individual. While BVs contain a single nucleocapsid, the AcMNPV ODVs contain multiple nucleocapsids that are enveloped together ODVs are generated during the “very-late” phase of infection and are embedded in a crystalline matrix made from the viral polyhedrin protein to form so-called “polyhedra” or “occlusion bodies” (OBs) in the nuclei of infected cells. After the death of the infected insect, the OBs are released into the environment, where their integrity is retained by the polyhedrin crystalline matrix, which protects the progeny virus from degradation by environmental factors, such as temperature and to a certain extent, UV-light. Upon ingestion of OBs and the release of the ODVs in the insect’s midgut, the next individual becomes infected. The BV are also the type of virus needed for systemic infection of the insect cells in culture and are therefore essential for production of rAAV in insect cells.

AcMNPV has a circular, double-stranded (ds) DNA genome with a size of approximately 134 kbp.^72^ The individual BV virions are rod-shaped, contain a single supercoiled DNA molecule and are 250–300 nm in size.^73^ During an infection with wt AcMNPV, there are two very-late phase genes, *p10* and polyhedrin (*polh*), expressed at high levels from their respective promoters. Whereas polh is the major structural component of OBs,^74^ P10 is a non-structural protein that facilitates their proper maturation.^75^^,^^76^ While P10 is not essential for OB formation, its absence results in structurally abnormal OBs.^75^^,^^77^ P10 and polh are neither required for BV production nor baculovirus propagation in insect cell culture.^76^^,^^78^ Both the p10 and polh promoters can therefore be used to drive high level expression of exogenous genes in cultured insect cells and are commonly employed in the BEVS.^79^

Through the incorporation of an antibiotic resistance gene and a bacterial origin of replication, the AcMNPV genome can be maintained in *E*. *coli* as a single-copy “bacmid” and easily edited with techniques, such as lambda red recombineering.^80^^,^^81^^,^^82^^,^^83^ Introduction of transgenes is nowadays typically done at the *polh* locus through attTn7-mediated transposition in *E*. *coli* or homologous recombination during co-transfection with a transfer plasmid on insect cells,^84^^,^^85^ though there are other options as well, including other loci for transposition, Gateway cloning, Cre-loxP site-specific recombination and Gibson assembly.^86^^,^^87^^,^^88^^,^^89^

## rAAV production

### Briefly: rAAV production in mammalian cells

During rAAV vector generation in mammalian cells the helper virus genes provide a multitude of functions. Mammalian helper viruses have been identified from the herpesvirus, AdV, papillomavirus families, as well as parvovirus human bocavirus 1 (HBoV1).^1^^,^^90^^,^^91^^,^^92^ The specifics of interactions between AAV and several helper viruses during production in mammalian cells is extensively reviewed in Meier et al.^40^ With AdV as an example of a helper virus, the minimal required AdV components are E1, E2A, VA RNA and E4 (Figure 3A).^93^^,^^94^^,^^95^^,^^96^^,^^97^ These essential helper functions mostly act to activate intrinsic AAV promoters for *Rep* and *Cap* expression (E1A, E2A), promote second strand synthesis (E1B, E4), aid in *Rep* mRNA splicing (E2A), and inhibit host cell immune responses and prevent AAV degradation (E4, VA RNA). Additionally, E1A drives the host cell into the DNA synthesis (S-) phase. Once in that phase, AAVs Rep78 can arrest the cell cycle.^98^^,^^99^ Commonly, rAAVs are produced in human embryonic kidney 293 cells (HEK293) through triple co-transfection with plasmids encoding the E2A, VA RNA, and E4 helper functions, AAV *Rep* and *Cap*, and the ITR-GOI (Figure 3A). Upon their generation, HEK293 cells were transformed with sheared AdV5 DNA and made to express the E1 gene, therefore it is not required to supply E1 in a helper plasmid when using these cells.^100^

**Figure 3:**
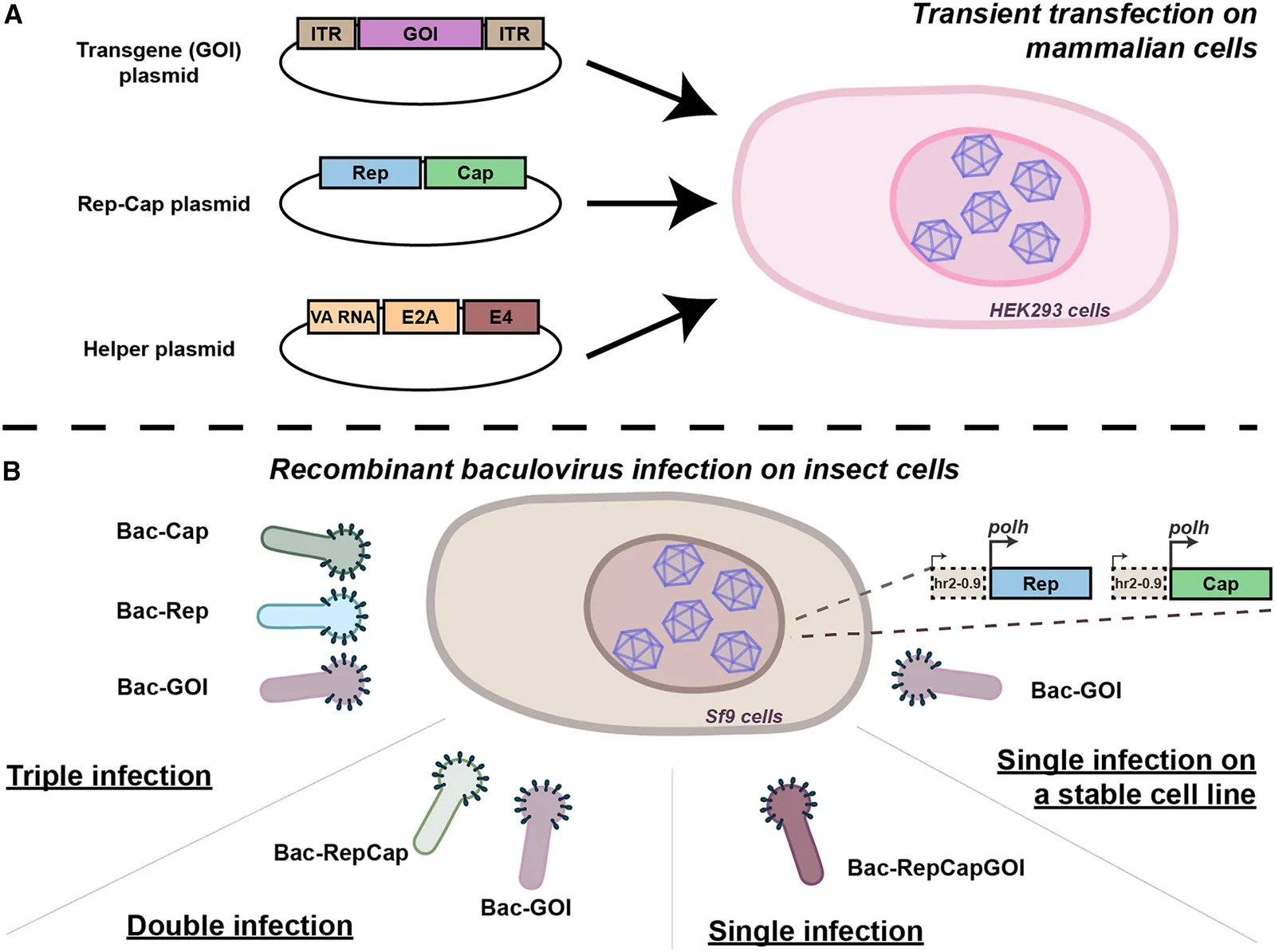
Overview of rAAV production strategies on mammalian and insect cells (A) Production in mammalian (HEK293) cells through a transient co-transfection with a GOI plasmid, Rep-Cap plasmid and plasmid containing AdV helper genes. (B) Production in (Sf9) insect cells with recombinant baculoviruses using a triple, double or single baculovirus infection to produce rAAV. (HEK293: Human embryonic kidney cells; ITR: inverted terminal repeat; GOI: gene of interest; Sf9 = Spodoptera frugiperda 9; hr: homologous repeat; polh: polyhedrin)

HSV-1 is another human virus that can be used as a helper virus for rAAV vector production in HEK293 cells.^4^ The HSV-1 helicase-primase complex, consisting of UL5, UL8, and UL52, and ICP8 protein (*UL29*) are all essential for rAAV replication.^90^ These proteins functionally replace AdV DNA replication helper functions by binding to Rep78 and AAV ssDNA, hence promoting AAV DNA replication.^101^ Although not essential, the HSV-1 immediate-early transcriptional regulators ICP0 (*RL2*) and ICP4 (*RS1*) were found to enhance AAV *Rep* and *Cap* expression.^102^ HSV-1 DNA polymerase activity is not required for AAV production, indicating that HSV-1 supports AAV replication primarily by providing replication-supportive and regulatory functions rather than directly performing genome synthesis.^90^ With HSV-1 as a helper virus, AAV2 replication was established to mostly takes place in the S/gap 2 (G2) phase.^103^

### Production in insect cells

Production of rAAV in insect cells differs fundamentally from mammalian platforms, as AAV is not known to naturally infect insects and was therefore not initially expected to be able to replicate in this environment. To circumvent the expected inactivity or suboptimal activity of native AAV promoters when producing AAVs with the BEVS, the *Rep* and *Cap* cassettes were cloned downstream of baculoviral promoters. The first demonstration of functional AAV *Cap* expression in insect cells was reported by Ruffing et al.^104^ who produced the three VP1, VP2, and VP3 from *polh* promoter copies present on promoters in three separate baculoviruses and demonstrated empty capsid assembly in Sf9 cells. While the suboptimal VP stoichiometry obtained with this method also led to aggregation of unassembled VP proteins, the study did establish that AAV capsid assembly is possible in insect cells.

A decade later, the first production of genome-containing rAAV particles using the BEVS was published (Figure 3B).^10^ In the study, *Rep*, *Cap*, and ITR-GOI were supplied via three separate baculoviruses. This work demonstrated functional *Rep* activity in insect cells for the first time. Because native AAV promoters and introns are poorly functional in insect cells, Rep78 and Rep52 were expressed from a truncated AcMNPV immediate-early 1 (IE1) and the *polh* promoter, respectively, while Cap expression was driven by a second copy of the *polh* promoter and regulated through non-canonical start codons to enable “leaky scanning” to allow expression of all three VPs.^105^^,^^106^ Genetic stability of the recombinant baculovirus was not assessed in the original study, but subsequent work revealed *Rep* gene instability after two to four passages, likely due to sequence identity between Rep78 and Rep52 that may have facilitated intramolecular homologous recombination.^107^^,^^108^ Despite drawbacks, including empty capsid formation and the requirement for triple co-infection, the study by Urabe et al.^10^ established BEVS as a promising platform for rAAV production, prompting further optimization in subsequent years.

Key improvements to the BEVS-rAAV production platform reduced the number of required baculoviruses from three to two by co-expressing *Rep* and *Cap* from a single baculovirus (Figure 3B).^11^^,^^12^ Chen addressed instability and poor expression associated with the native Rep intron in insect cells through introduction of artificial introns into the *Rep* and *Cap* coding sequences, enabling expression of Rep78 and Rep52 from a single orf while preventing large homologous repeats.^12^ This design showed genetic stability through passage 5. Smith et al.^11^ instead employed a bifunctional mRNA strategy based on leaky scanning to express Rep78 and Rep52 by extending on a mechanism natively applied to *Cap* expression in wtAAV. This recombinant baculovirus maintained consistent *Rep* and *Cap* expression through passage 6, after which expression declined. Together, these studies demonstrated improved genetic stability, higher rAAV titers, and reduced system complexity, all of which significantly enhanced the feasibility of large-scale rAAV production with the BEVS.

Although the reduction to only two baculoviruses improved robustness, further simplification was pursued to facilitate low-MOI infection strategies, which would reduce virus input, stock virus passaging cycles, and production costs. Wu et al.^109^ first demonstrated rAAV production from a single baculovirus by combining *Rep*, *Cap*, and an ITR-GOI within one vector using a leaky scanning strategy (Figure 3B). However, genetic instability emerged after passage 5. Galibert et al.^110^ subsequently reported a single-baculovirus system with *Rep* and *Cap* expressed from *polh* and *p10* promoters, respectively, and the transgene cassette (ITR-GOI) integrated at a separate locus in the baculovirus genome, demonstrating rAAV production in shake flask and bioreactor settings. In parallel, alternative approaches based on stable producer or packaging cell lines were developed, most notably the “OneBac” system, in which *Cap* and *Rep* are inducibly expressed and rAAV production is achieved by infection with a single baculovirus carrying the ITR-GOI.^16^^,^^18^^,^^111^

## Importance of host cell environment

Insect cells infected with recombinant baculovirus provide an altered intracellular environment for rAAV replication compared to production on mammalian cells, yet the system still supports *Cap* and *Rep* expression, ITR-dependent replication/processing, and capsid assembly. Apparently, baculovirus and/or insect-cell derived factors appear to substitute for adenoviral helper functions, although the exact identities and mechanisms are incompletely mapped. Insect cell lines used for the production of recombinant proteins with the BEVS are commonly derived from *Spodoptera frugiperda* (e.g., Sf9, Sf21) or *Trichoplusia ni* (Hi5). While the application of these cell lines for the production of therapeutics has been demonstrated,^112^ Sf9 cells are generally favored and more widely applied, mainly due to their increased capacity of progeny baculovirus production compared to Hi5,^113^ and increased tolerance to culture condition variations compared to Sf21.^114^ The use of Hi5 cells for rAAV production has been documented once before, where no significant differences in vector yields were reported in a comparison between Sf9 and Hi5 cells.^115^ In this review we focus on rAAV production in Sf9 cells, since it is the most predominantly used insect cell line for this purpose.^116^

Although unexpected due to the stark differences between mammalian and insect cells, the activity of native AAV promoters has been detected during the attempted production of wtAAV2 in insect cells.^117^ In the study, the full genome of AAV2 was inserted into a bacmid, which was used to transfect Sf9 cells. Apparent native p5 and p19 rep promoter activity in insect cells caused excision of the transgene sequence from the baculovirus during earlier phases of infection. This observation provides evidence that baculovirus infection creates an environment permissive for at least partial activation of AAV regulatory elements. This supports the broader notion that baculovirus infection supplies helper-like functions for AAV in insect cells, not only by remodeling host DNA replication pathways or expressing *Cap* and *Rep* from baculoviral promoters, but also by enabling activity of AAV *cis*-regulatory elements that would otherwise be expected to be inactive.

AAV is a mammalian virus and naturally replicates in vertebrate hosts at a temperature around 37°C. In contrast, recombinant AAV production using the baculovirus-insect cell system is routinely performed at 27–28 °C, the optimal temperature for baculovirus infection and insect cell metabolism.^118^^,^^119^ This apparent temperature discrepancy likely reflects the permissive replication environment created by baculovirus infection, which drives strong expression of AAV components and sustains active host DNA metabolism at the lower temperatures.

As mentioned before, Rep78 itself can modulate the cellular state to arrest the cell cycle at the S-phase, although it requires helper functions that induce the S-phase in the first place.^98^ Despite Rep78 causing a cell cycle arrest here, AAV genome replication is reported to increase when the cell cycle is arrested in the mitotic (M) or gap 2 (G2) phase.^120^^,^^121^ Baculoviruses are known to induce extensive host cell reprogramming to support viral DNA replication, and they can induce and arrest the cell cycle in the M, G2 or S-phase.^122^^,^^123^ Baculovirus infection drives infected insect cells into a replication-competent state characterized by sustained activity of host DNA replication and repair pathways.^124^^,^^125^ This baculovirus-induced cellular state likely provides the replication machinery exploited by AAV Rep to drive vector genome amplification, functionally substituting for adenoviral E1A-dependent host activation. It remains unclear which baculovirus gene products or infection-induced host responses are specifically required for AAV replication, and whether helper activity arises from discrete baculovirus factors or from general adjustment of the host replication environment.

Efficient AAV DNA replication requires host replication factors, including DNA polymerase δ, cellular replication factor C (RFC), proliferating cell nuclear antigen (PCNA), and the minichromosomal maintenance (MCM) helicase complex, together with the viral Rep protein.^126^ Depletion of MCM components or DNA polymerase δ strongly impairs AAV genome replication, underscoring AAV’s reliance on the host replication machinery.^126^ PCNA is conserved among eukaryotes and is an essential processivity factor for DNA replication, as well as involved in cell cycle control, DNA repair and chromatin remodeling.^127^ In mammalian cells, viral helper functions benefit AAV by upregulating these host replication factors.^40^^,^^128^^,^^129^ In baculovirus-mediated rAAV production, no direct homologs of adenoviral helper genes have been identified. However, baculoviruses do encode for a PCNA homolog (*ac49*),^130^^,^^131^ as well as a DNA polymerase (*ac65*) belonging to the same B-family type of polymerases where DNA polymerase δ belongs to.^132^ Possibly, these baculovirus homologs are substituting for the AdV helper functions that recruit these factors during production on mammalian cells.

Lastly, to point out the key role the host cell environment plays, there are reports of AAV replication occurring in mammalian cells without a helper virus or helper virus genes present, although this required extreme changes in cellular environment. Yakobson et al.^133^ demonstrated successful AAV2 replication in Chinese hamster (OD4) cells after inducing DNA-damage through pre-treatment with hydroxyurea, polyamine depletion or mitotic detachment. Additionally, induction of DNA-damage through irradiation with UV light had the same effect.^134^ The treatments caused an arrest in the S-phase for the majority of the cells, which is when the cellular environment is optimal for most autonomous parvoviruses to replicate.^135^ Despite only detecting AAV replication in few cells of the treated cell population, the findings do highlight the importance of the correct cellular environment during AAV replication. Whether this approach could have the same effect in insect cells remains to be determined.

### Plausible baculovirus-AAV interactions: Candidate baculovirus genes

In mammalian systems, AAV replication strictly depends on adenoviral helper functions that modulate host transcription, DNA replication, and DNA damage responses (DDRs). Similar to the production with mammalian helper viruses, replication of AAV in the BEVS is unlikely driven by a single helper factor, but permitted by a modified cellular state induced by baculovirus infection.^136^ The mammalian helper viruses and baculoviruses are evolutionary distinct, therefore no direct sequence homologs of AdV or HSV helper genes exist in the baculovirus. However, identifying functional homologs that recreate an equivalent nuclear environment is a rational strategy (Table 1). When producing rAAVs with the BEVS, some helper functions aiding in the activation of intrinsic AAV promoters can be circumvented by driving *Rep* and *Cap* expression with baculoviral promoters as explained before. In this section, we compare functional homology between AdV, HSV, and baculoviral helper functions in the context of AAV replication without the use of baculovirus promoters for *Rep* and *Cap* expression, for a more complete overview. The proposed candidate baculoviral helper genes are summarized in Table 1, and discussed in the following sections.

**Table 1:** Putative baculovirus helper genes for AAV replication based on predicted functional homology with AdV or HSV helper viruses

Helper function	Mammalian virus helper genes or proteins	Putative baculovirus functional homologs
Transcriptional activation/nuclear reprogramming	E1A (AdV); ICP4, ICP0 (HSV)	IE-1 (ac147), IE-2 (ac151), PE38 (ac153)
Replication-permissive host state/cell-cycle coordination	E1A (AdV)	PCNA (ac49)
DNA unwinding (helicase activity)	UL5/UL8/UL52 (HSV)	p143 helicase (ac95)
ssDNA binding and stabilization	E2A (AdV); ICP8/UL29 (HSV)	LEF-3 (ac131)
DNA priming/primase activity	Host Pol α recruitment (AdV); UL52 (HSV)	LEF-1 (ac14), LEF-2 (ac58)
DNA processing/recombination	E4 gene products (AdV); UL12 (HSV)	ALK-EXO (ac133)
Late-stage genome handling/packaging environment	UL25/UL32 (HSV)	VLF-1 (ac77)
DNA damage response modulation	E4orf6/E1B55K (AdV); ICP0 (HSV)	LEF-7 (ac96)
Stress response/translation competence (PKR pathway)	VA RNA (AdV); US11 (HSV)	PK2 (ac123)

AdV, adenovirus; HSV, herpes simplex virus; IE, immediate early; ac, AcMNPV open reading frame; LEF, late expression factor; ALK-EXO, alkaline exonuclease; VLF-1, very-late factor 1; PKR, protein kinase R; PK, protein kinase.

### Transcriptional reprogramming

A common feature of AAV helper viruses is the functioning of early regulatory proteins that reprogram host transcription. In AdV, this role is fulfilled by E1A, while in HSV it is mediated primarily by ICP0, ICP4 and ICP22.^93^^,^^137^ Baculoviruses encode for comparable transcriptional regulators, including immediate-early 1 (IE-1), IE-2, and PE38, which together mediate early and early to late viral gene expression and broadly remodel host transcriptional activity.^138^^,^^139^ IE-1 is the major immediate-early transactivator, plays a crucial role in the switch to replication, and is essential for initiating the baculovirus transcriptional cascade, while IE-2 and PE38 further modulate viral and host gene expression.^140^ Although these proteins share no sequence homology with AdV or HSV regulators, their ability to establish a virus-dominated transcriptional environment suggests functional equivalence that may indirectly enhance AAV *Rep* and *Cap* expression.

## DNA replication support

AdV E2A is a ssDNA-binding protein, which enhances the stability and processivity of the AAV genome.^94^ With HSV as a helper virus, the helicase-primase complex (UL5/UL8/UL52) and ICP8 perform analogous roles.^101^ Baculovirus encodes a set of core DNA replication proteins that may functionally parallel these activities. These include the p143 helicase, the late expression factor 1 (LEF-1) and LEF-2 primase complex, and the LEF-3 ssDNA-binding protein.^73^ LEF-1 is a DNA primase essential for baculovirus DNA synthesis, and works with LEF-2 acting as a cofactor.^141^ LEF-3 binds to and stabilizes ssDNA replication intermediates.^142^ Together, these factors may support AAV replication by stabilizing Rep-generated DNA structures and through primase activity. Additionally, baculovirus encode a PCNA, a delayed-early gene product involved in DNA synthesis and repair, as well as cell-cycle coordination.^143^ This protein may further support a cellular environment conducive to AAV genome replication.

### DNA processing and genome maturation

Beyond replication, helper viruses provide DNA processing activities that facilitate genome maturation. In AdV, E4 gene products promote AAV second strand synthesis and RNA processing,^6^ and HSV encodes an alkaline exonuclease (UL12), which processes DNA replication intermediates.^102^ Baculovirus encodes an alkaline exonuclease (ALK-EXO) involved in viral DNA recombination and processing, and it is a close functional analog to the compared proteins in AdV and HSV, representing a strong candidate for involvement in AAV genome processing through circularization and/or maturation.^144^

Another candidate is very-late expression factor 1 (VLF-1), which is a DNA-binding protein required for very late gene expression and viral DNA packaging.^145^^,^^146^ Although primarily characterized as a transcription factor, the DNA binding properties of VLF-1 and its role in genome handling raise the possibility that it modulates replication or genome organization in ways that could influence AAV packaging efficiency.^145^^,^^147^

### Modulation of host antiviral responses

Manipulation of the cellular DDR is an important helper function of mammalian AAV helper viruses. AdV actively suppresses DDR signaling through E4orf6/E1B55K, while HSV-1 employs ICP0 to disrupt intrinsic antiviral defenses.^148^ Additionally, AdV VA RNAs also aid in mediating host defenses through inhibition of the antiviral protein kinase R (PKR) pathway.^149^ Baculovirus infection effectively manipulates the host cells’ DDR response to promote viral DNA replication and propagation through the activity of LEF-7.^150^^,^^151^ Additionally, baculovirus encodes a protein kinase 2 (PK2), which is involved in evading PKR-mediated protein shut off mechanisms and mitigating stress-induced antiviral responses, thereby supporting viral DNA replication and protein synthesis.^152^^,^^153^

## Suggested experimental approach to verifying interactions

### Transcriptional and proteomic time course experiments

Transcriptomics has been performed on the Sf9 cells during production of rAAV with the BEVS before with intent to identify insect cell population variability during production and its effect on product yield and quality.^154^^,^^155^ A transcriptional and proteomic time-course analysis can also support identification of baculovirus helper functions by temporally linking baculovirus gene expression with stages of AAV replication and packaging. Baculovirus infection follows a tightly regulated cascade of immediate-early, early, late, and very-late gene expression, each associated with specific cellular processes such as DNA replication, virus transcriptional control, and virion assembly.^79^^,^^156^ RNA-seq performed at different hours post-infection allows for the simultaneous quantification of baculovirus, AAV, and host transcripts (Figure 4A). By doing so, genes whose expression or protein abundance precedes or coincides with, for example Rep accumulation, AAV and baculovirus genome replication, capsid synthesis or virion assembly, can be investigated as candidate helper factors. Integration of (quantitative) proteomics data may be particularly helpful, as it captures post-transcriptional regulation and protein dynamics that are not reflected at the RNA level. This addition could enable discrimination between genes that are merely co-expressed and those whose gene products that interact with AAV gene expression and influence replication.

**Figure 4:**
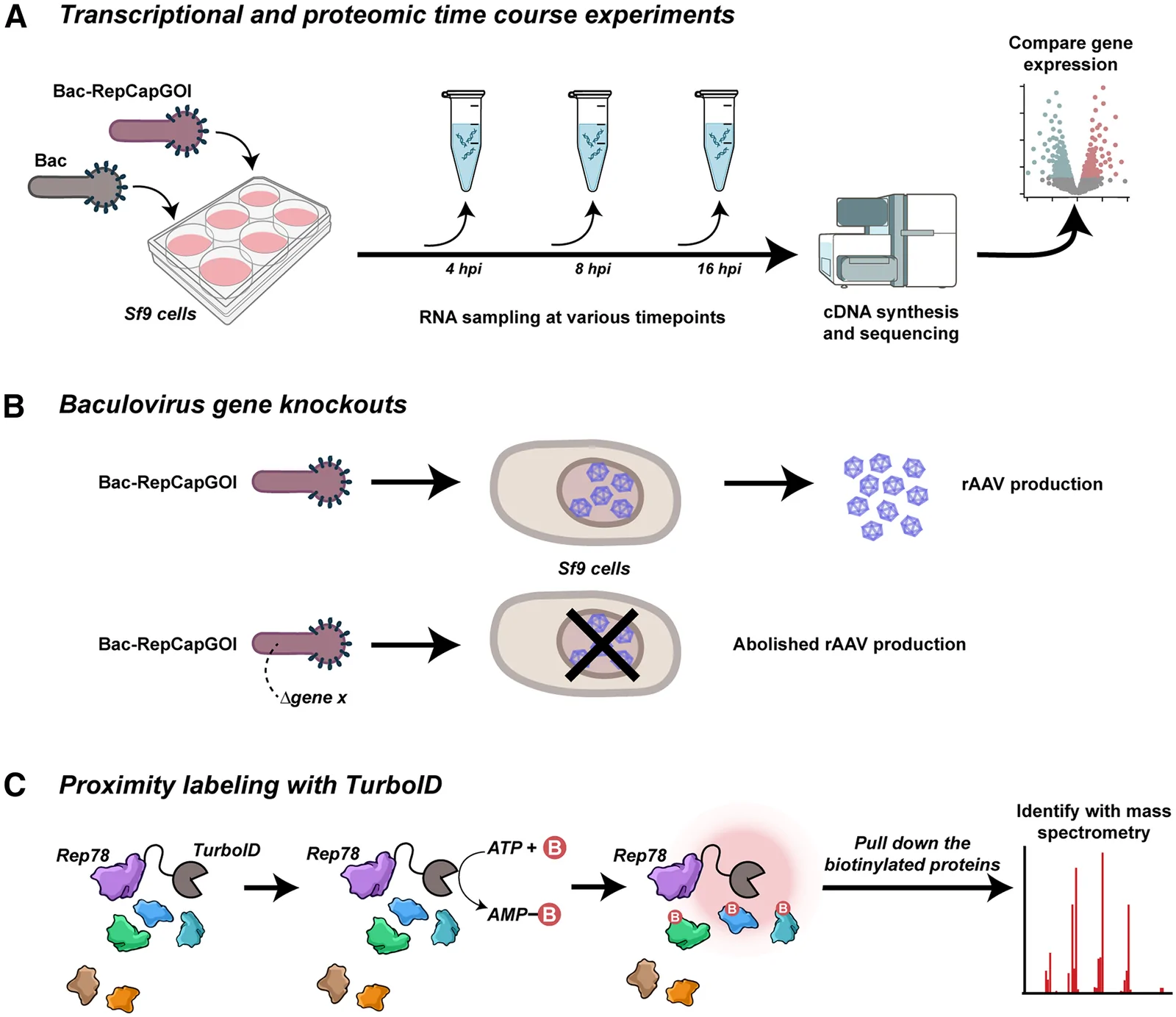
Suggested experimental approaches for identifying baculovirus and (r)AAV interactions (A) Through infection of Sf9 cells with baculoviruses that contain or lack (some or all) AAV transgenes and harvesting RNA samples at separate timepoints for RNA-sequencing, differentially expressed genes can be identified. These differential expressions can point into the direction of interactions between AAV, baculovirus and Sf9 cells. (B) By generating baculovirus deletion mutants, the impact of specific deletions on the replication and generation of rAAV can be tested. (C) TurboID can be fused to an AAV (e.g., Rep78) or baculovirus protein to detect proteins that are in close proximity and may be interacting (in)directly with the labeled protein. Upon biotin addition, ATP and biotin are converted by the TurboID enzyme into biotin-AMP, which covalently labels proximal endogenous proteins. These can be pulled down with streptavidin beads in an affinity purification assay, and subsequently identified mass spectrometry. (hpi, hours post infection; cDNA, complementary DNA)

### Functional necessity screen: Baculovirus gene knockouts

To directly assess which baculovirus genes are functionally required for efficient rAAV replication and packaging, targeted baculovirus gene deletion mutants can be generated and evaluated for their ability to support AAV production in insect cells such as Sf9 (Figure 4B). Comparing rAAV yields, genome replication, and capsid assembly across individual deletion mutants would provide strong evidence for helper activity.

A major limitation of this approach is that many baculovirus genes are essential for baculoviral DNA replication, late gene expression, or BV formation, complicating interpretation when deletions compromise baculovirus viability itself.^125^^,^^157^^,^^158^ To address this, conditional strategies may be employed, such as inducible or partial knockdowns, or the use of complementing insect cell lines that stably express the candidate baculovirus gene. This “from-the-ground-up” complementation approach can decouple the role of a gene in baculovirus propagation from its potential helper function in AAV biology.

Generating baculovirus deletion mutants with defects in defined phases of infection can be a useful strategy to investigate whether AAV genome replication and capsid formation depend on specific baculoviral functions. Examples could be the deletion of *lef-1* (*ac14*) or *vlf-1* (*ac77*).^145^^,^^159^ The *lef-1* gene encodes a DNA primase required for baculovirus DNA replication, and deletion results in viruses that fail to progress into late and very late phases of infection.^160^ Deletion of *vlf-1* abolishes very late gene expression without preventing earlier stages of viral replication.^146^ By supplying the AAV Rep, Cap and ITR-GOI *in trans* with (non-)baculoviral promoters unaffected by *vlf-1* impairment, the impact of specific baculoviral gene expression defects on AAV genome replication and capsid formation can be assessed with a reduced dependence on baculovirus late or very late gene expression. Rep-dependent AAV genome replication can be quantified by qPCR, while capsid assembly can be monitored using an ELISA assay.

Rescue experiments are critical to validate specificity. If the deletion of a candidate gene reduces rAAV yield or alters replication phenotypes, re-introduction of the gene via a helper baculovirus or plasmid should restore AAV production. Such genetic complementation strengthens causal inference and helps rule out indirect effects stemming from unintended baculovirus defects.

### Proximity labeling

Proximity-dependent labeling approaches offer a powerful, unbiased strategy to identify physical and spatial associations between AAV proteins and baculovirus or host cell factors (Figure 4C). Fusion of proximity labels, for example with BioID or (split-)TurboID, to AAV proteins would enable biotinylation of neighboring proteins during active infection.^161^^,^^162^^,^^163^^,^^164^ After affinity purification, mass spectrometry needs to be performed to identify the interacting proteins. This approach is particularly attractive for application in the BEVS-rAAV system, where transient or weak interactions may be difficult to capture by conventional co-immunoprecipitation methods. Proximity labeling can reveal candidate helper proteins involved in replication complex assembly, genome packaging, nuclear trafficking, or capsid maturation. Complementary to the proximity labeling, other localization studies using fluorescence microscopy or (cryo-)electron microscopy could further refine spatial context, for example by mapping Rep-associated replication compartments relative to baculovirus replication complexes.

## Conclusions and future prospects

rAAV vectors have a promising therapeutic potential for many genetic disorders. Since its first description in 1983, the BEVS has been developed into a dependable biotechnological platform for the production of various recombinant proteins.^71^^,^^79^ Despite early initial descriptions of rAAV production with the BEVS in 2002, it took several years of development for the application to experience the process development and optimization needed to convert into the promising platform it has become today.^10^^,^^165^ With rAAV making up for approximately a third of the vectors used in clinical trials for *in vivo* gene therapy,^166^ and the BEVS being continually evolved for vector production, gaps in the fundamental understanding of the interaction between these two viruses became more apparent. It is unclear which baculovirus genes are required, supportive, or dispensable for directly or indirectly supporting (r)AAV replication, whether AAV genome replication centers spatially or temporally overlap with baculoviral replication factories, and which steps in the AAV life cycle constitute the principal bottlenecks in BEVS. Moreover, the role of insect host cell factors and the effect of different post-translational modifications occurring during production in insect cells on the *in vivo* vector potency are important points of study.^167^ These unknowns are reflected in persistent challenges such as variable genome packaging efficiencies, serotype-dependent productivity, and empty capsid formation percentages.^168^^,^^169^^,^^170^^,^^171^

Addressing these questions will advance our mechanistic understanding of the helper virus complementation by the baculovirus, while also enabling the rational engineering of improved BEVS-based rAAV production systems. The determination of baculovirus helper functions may ultimately allow the development of minimal helper modules or reduced baculovirus genomes, aiding to enhance yield, consistency, and product quality. The application of synthetic biology for the minimization of baculovirus genomes provides an opportunity to tailor baculovirus vectors for improved helper function while reducing unnecessary viral burden.^172^^,^^173^^,^^174^ Multi-omics approaches, combining transcriptomics, proteomics, and metabolomics, can be applied to identify insect cell host factors and baculovirus gene products that influence (r)AAV genome replication, genome packaging, and capsid assembly, thereby providing new targets for producer cell line and process engineering.^154^^,^^155^^,^^175^ Together, these approaches may enable the transition from empirical optimization toward rational engineering of BEVS platforms for rAAV production.

## Acknowledgments

This work was funded by VectorY Therapeutics. We thank Jelmer Vroom from WEMC for the TEM images, Hans Bastiaansen for assistance in obtaining the samples for TEM, and Astrid Bryon for providing the basis of the script used for the phylogeny analysis.

## Author contributions

L.A.d.J. conceived the research, reviewed literature, wrote and edited the manuscript. M.M.v.O. acquired the funding, wrote and edited the manuscript. G.P.P. contributed to the conception of the research, supervised the project, wrote and edited the manuscript.

## Declaration of interests

The authors declare no competing interests.

## Supplemental information

Document S1. Table S1

Document S2. Article plus supplemental information
