# Supplementary material for: Baculovirus helper functions during recombinant adeno-associated virus production in insect cells for gene therapy applications
Source: Mol Ther Adv. 2026 Oct 26;34(4):201876. doi: 10.1016/j.omta.2026.201876 (PMC13770656; doi:10.1016/j.omta.2026.201876)
Supplement: Supplementary file 1 [file mmc1.pdf]

OMTA, Volume *34*

Supplemental information

Baculovirus helper functions during recombinant adeno-associated virus production in insect cells for gene therapy applications

Linda A. de Jong, Monique M. van Oers, and Gorben P. Pijlman

Supplemental

Table S1. NCBI GenBank accession numbers of the viral protein sequences used to construct the phylogenetic tree. Accession numbers of the nonstructural protein 1 (NS1) protein sequences from the viruses that were used to build the phylogenetic tree in Figure 1. Extracted from NCBI (https://www.ncbi.nlm.nih.gov) on 27 January 2026.

**GenBank Accession number**	**Description**	**Virus**
NP_852780.1	rep protein	Avian adeno-associated virus ATCC VR-865
ABZ10811.1	nonstructural protein	Adeno-associated virus 13
AAT46336.1	nonstructural protein	adeno-associated virus 10
AAT46338.1	nonstructural protein	Adeno-associated virus 11
ABI16638.1	Rep78	Adeno-associated virus 12
PU583190.1	Rep78	Adeno-associated virus 9
AAB95449.1	nonstructural protein Rep78	Adeno-associated virus - 6
YP_010086821.1	Rep	Murine adeno-associated virus 2
YP_010086819.1	Rep	Murine adeno-associated virus 1
YP_009507366.1	Rep78	California sea lion adeno-associated virus 1
YP_003858571.1	rep protein	Bat adeno-associated virus YNM
YP_680423.1	Rep 78 protein	adeno-associated virus 2
YP_077182.1	rep protein	Avian adeno-associated virus strain DA-1
YP_077179.1	nonstructural protein	adeno-associated virus 8
YP_077177.1	nonstructural protein	adeno-associated virus 7
YP_068410.1	REP protein	Muscovy duck parvovirus
YP_068408.1	DNA binding trs helicase	adeno-associated virus 5
YP_024970.1	Rep	Bovine adeno-associated virus
NP_049541.1	nonstructural protein	Adeno-associated virus - 1
NP_044926.1	rep	Adeno-associated virus - 4
NP_043940.1	nonstructural protein	Adeno-associated virus - 3
NP_043514.1	REP	Goose parvovirus
ABC69725.1	Rep78	Caprine Adeno-associated virus-Go.1
QPN96232.1	nonstructural protein	Bearded dragon parvovirus
YP_068093.1	non-structural protein 1	Snake adeno-associated virus
